# Water assisted biomimetic synergistic process and its application in water-jet rewritable paper

**DOI:** 10.1038/s41467-018-07211-z

**Published:** 2018-11-16

**Authors:** Guan Xi, Lan Sheng, Jiahui Du, Jinyan Zhang, Minjie Li, Hongze Wang, Yufei Ma, Sean Xiao-An Zhang

**Affiliations:** 1 State Key Laboratory of Supramolecular Structure and Materials, College of Chemistry, Jilin University, 130012 Changchun, China; 2 College of Chemistry, Jilin University, 130012 Changchun, China; 3 Lucky Healthcare Limited Liability Company, 071054 Baoding, China

## Abstract

The colour of water-jet rewritable paper (WJRP) is difficult to be expanded via single hydrochromic molecule, especially black. Here, inspired by the amazing phenomenon of bound-water in cells enabling various biological transformations via facilitating synergistic inter-/intra-molecular proton transfer, we present a simple strategy toward WJRP based on binary systems containing less-sensitive acidochromic dyes and mild proton donors (or developers). With such a binary system containing commercial black dye as the colouring agent, benzyl 4-hydroxybenzoate as the developer, and biomimetic bound-water as proton-transferring medium, we successfully achieve the long-awaited black WJRP. Printed images on such WJRP have excellent performances and long retaining time (>1 month). In addition, the robustness, durability and reversibility of WJRP could be increased distinctly by using polyethylene terephthalate as substrate. This strategy significantly expands hydrochromic colours to entire visible range in an eco-friendly way, which opens an avenue of smart materials for practical needs and industrialization.

## Abstract (web summary)

Water based inks used for water-jet rewritable paper (WJRP) are an environmental friendly alternative to conventional printing, but black colour in WJRP could not be realized so far. Here the authors demonstrate black as well as other colour WJRP based on binary systems containing less-sensitive acidochromic dyes and mild proton donors.

## Introduction

**Fig. 1:**
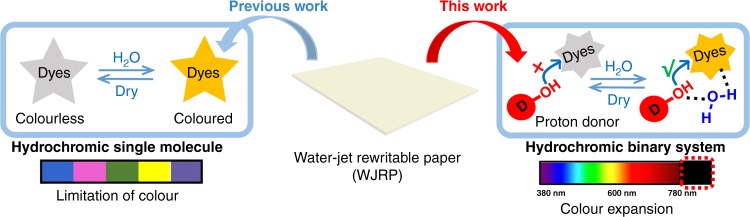
Schematic illustration of different mechanism for water-jet rewritable paper (WJRP) between this work and our previous work. Mechanism of WJRP showing colour based on the hydrochromic single molecule in our previous work and the binary system in this work after addition of water

Nowadays, most paper and ink are discarded after being used once, leading to severe solid waste, water pollution, global haze, deforestation, and desertification^1^. Rewritable paper, which is repeatedly usable, is an alternative approach to alleviating these environmental problems. To date, several approaches have been taken in this field, various impressive materials responsive to light^2–5^, heat^6–8^, stress^9–11^, solvent^12–14^, and acid/base^15,16^ have been developed for potential rewritable paper^17^ and inkless printing. Among them, water has been regarded as one of the most attractive stimulus given its promising advantages of clean, green, low cost, convenience, and compatibility with current ink-jet printing. So far, a few interesting studies have demonstrated its feasibility. Such as, certain periodically ordered photonic crystals embedded in hydrogels or polymer matrices^18–20^, which vary reflectance wavelengths depending on water content, and hydrochromic dyes, which can change their configuration and structure responding to water^2,21–25^, are both potentially applicable in water-jet rewritable paper (WJRP). Initial hydrochromic dyes based WJRP^22,24^ has demonstrated very promising potential with fast colouration, remarkable colour uniformity, and purity (Fig. 1, left). However, development of suitable hydrochromic dyes has been significantly held back from their commercialisation because of the following reasons: (1) most of such dyes are usually complicated in synthesis/preparation which is time-consuming and the dyes are expensive; (2) long-awaited water-sensitive black dyes for ideal display have not been achieved yet. These limitations are difficult to overcome using existing hydrochromic process and mechanism. Thus, it is highly desirable to develop long-anticipated black WJRP based on new switching mechanisms.

Inspired by thermochromic black display, that is, acidochromic black dye would accept the protons from thermo-proton-donors and change its colour by heating^26^, as well as that some key biochemical reactions involving proton delivery by water-networks^27,28^, we conjecture that it is possible to use bound-water among proton donors and acidochromic dyes to switch the dyes. Wherein, the inserted bound-water in the system might act as a proton transfer bridge and ion stabilizer to regulate the dyes’ colour switching.

Herein, in this work, a strategy toward ideal WJRP based on water assisted binary systems containing acidochromic dyes and proton donors (or developers), has been developed (Fig. 1, right). After systematic examination of proper black acidochromic dyes, developers, and their suitable concentration, the black WJRP based on the binary system is constructed, and their basic performances are explored. The binary system based WJRP has distinct advantages of realizing black, fast response, high contrast and long retaining time (>1 month). Related hydrochromic mechanism is further understood from the molecular level. In addition, this unique approach of colour tuning with common acid-responsive dyes will make a wide variety of colours possible for future WJRP. This will not only avoid traditional complicated synthesis/preparations and dramatically reduce related industrialization cost, but also make extension of ideal colour for the WJRP much easier, especially the black WJRP.

## Results

### Design and characters of WJRP

**Fig. 2:**
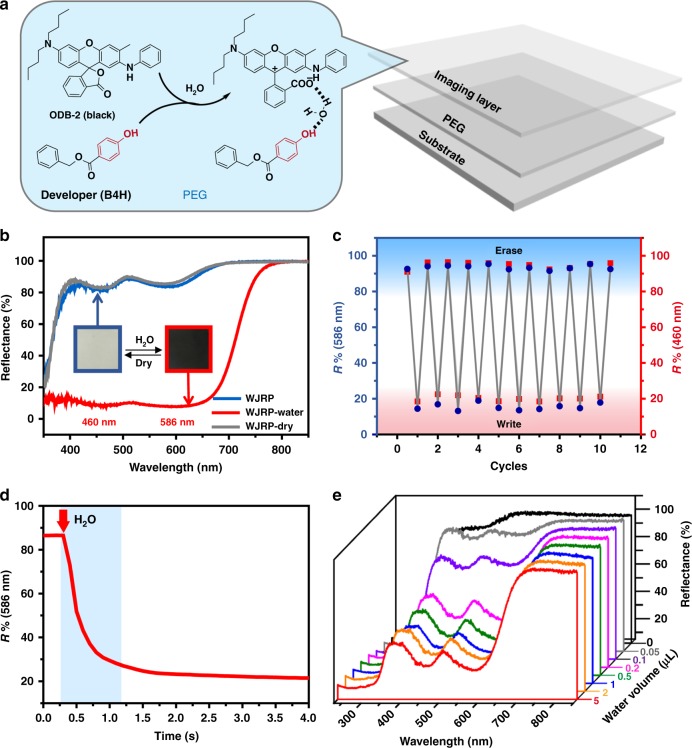
Structure and basic performances of WJRP. **a** The preliminary structure of WJRP and the composition of this hydrochromic system in the imaging layer of WJRP (black dashed line: H-bonding). **b** The reflective UV-vis spectra of the WJRP before and after addition of water as well as drying the WJRP. **c** Plots of the reflectivity at 460 nm (red dots) and 586 nm (blue dots), respectively versus the number of cycles as the WJRP is cycled through water spraying (write) and water removal (erase) by means of heating at 80 °C. **d** Time-dependent reflection variations of black WJRP at 586 nm during exposure to a water droplet (red arrow: the point of adding water). **e** The reflective UV–vis spectra of WJRP after exposure to different volume of water

To explore viability of our approach and solve reusable black display, a black leuco dye (ODB-2) and benzyl 4-hydroxybenzoate (B4H) were chosen as an example for proposed binary hydrochromic system to realize black WJRP. B4H was chosen as a developer for its proper hydrophobicity and proton-donating-ability. With reference to our previous work^22^, the black WJRP was constructed by a three-layer structure: a substrate at the bottom, a polyethylene glycol (PEG) layer in the middle and an imaging layer on the top (Fig. 2a). The substrate of WJRP is filter paper for its simple composition and good adsorption. PEG is utilized herein for efficient passivation of the paper’s hydroxyl groups via hydrogen-bonding. And the preliminary composition of the imaging layer for black WJRP is the binary system of ODB-2 and B4H dissolved in PEG to avoid microcrystalline aggregates. To achieve satisfactory black display after addition of water, the optimal molar ratio of ODB-2 to B4H on WJRP has been screened and confirmed as 1/4 (see Supplementary Fig. 1a,b). The colour of this initially-prepared WJRP was white with >80% reflectance in the absence of water (Fig. 2b, cyan line). After addition of water, it turned to black immediately and two broad peaks with almost identical reflectance intensity decreased below 10% (Fig. 2b, red line), nearly covering the entire visible region. This spectrum is almost the same with that of the paper based on ODB-2 alone in the same concentration upon addition of excessive amount of acid (see Supplementary Fig. 1c). It indicates that the colour intensity of WJRP induced by water is comparable to the acid-induced colour intensity. When water evaporated at room temperature or was quickly removed by heating, the paper could turn to its original white state and the reflection bands restored to the original position (Fig. 2b, grey line). These demonstrate that the binary system based WJRP is feasible and in accordance with our expectations.

To confirm reversibility of the WJRP, reflectance of WJRP at 460 nm and 586 nm (two main peaks of ODB-2 in zwitterionic form) were measured respectively, through writing with water and erasing with heating. It was found that no obvious decrease in colour intensity or contrast for both coloured and blank states of WJRP could be observed after more than 10 consecutive writing-erasing cycles (Fig. 2c). This indicates that such binary system based WJRP has excellent reversibility.

A critical factor, which needs to be considered in developing broader applications of WJRP, is their water sensitivity. Therefore, in situ spectral dynamics was applied to monitor the rate of water-promoted colourimetric response of the WJRP. A plot of reflectance intensity at 586 nm as a function of time following exposure to a water droplet is shown in Fig. 2d. The result reveals that significant colour change occurs within 1 s after water contacts the paper, that is, the colour appears instantly after a water-jet printer finishes printing. Moreover, only 0.5 μL water is sufficient to make a 3 mm^2^ area of the WJRP turn to black noticeably (Fig. 2e). All these suggest that the WJRPs based on the binary system are water sensitive enough for practical usage.

### The proposed hydrochromic mechanism of the WJRP

**Fig. 3:**
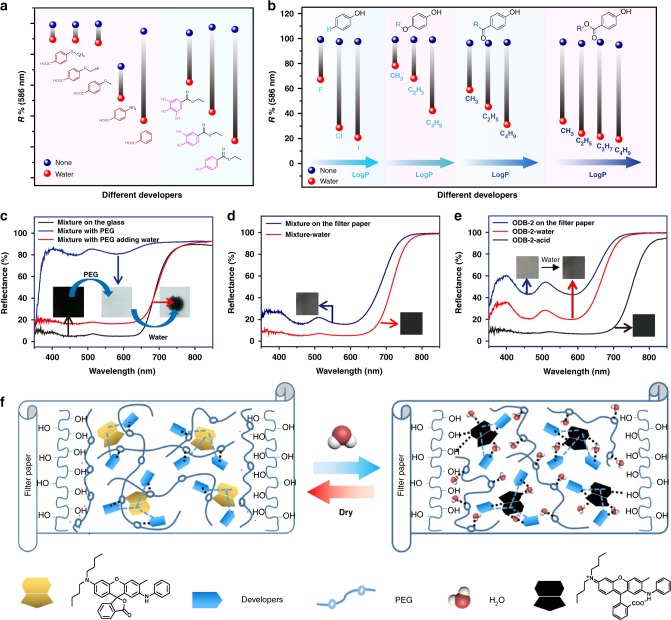
Function of each component in WJRP and the hydrochromic mechanism of WJRP. Variation of reflectance (*R*%) at 586 nm of WJRP integrated with ODB-2/developers (*n*/*n* 1/4) including **a** carboxylic acid derivatives and polyhydroxyls phenol derivatives and **b** developers with different logP before (blue dots) and after addition of water (red dots). **c** UV–vis reflection spectra of the mixture of ODB-2 and B4H on the glass before and after introduction of PEG and the mixture with PEG after adding water. **d** UV–vis reflection spectra of the mixture of ODB-2 and B4H on filter paper before and after addition of water. **e** UV–vis reflection spectra of ODB-2 alone dispersed on filter paper before and after addition of water as well as adding acid. **f** Schematic representation of WJRP based on the binary system writing with water and the erasing process (blue dashed line: supramolecular interaction, black dashed line: H-bonding)

To better understand the hydrochromic mechanism of this WJRP, it is crucial to figure out the function of each component including developers, dyes (ODB-2), PEG, water, as well as the paper substrate. Developers, as the proton donors, should have many candidates except for B4H. Carboxylic acids and polyhydroxy compounds in principle had strong ability to give out protons^29,30^ were expected to be good developers. Hydrochromic property of the WJRPs taking carboxylic acids as developers instead of B4H was explored firstly. To our surprise, after addition of water, most carboxylic acid derivatives were poor to develop colour for WJRPs (Fig. 3a, left). It was conjectured that strong intermolecular hydrogen bond interactions among the acids render them prone to self-aggregation^31^, resulting in phase separation between ODB-2 and the carboxylic acid derivatives. This conjecture was confirmed by the fact that respective crystals of ODB-2 and carboxylic acids were generated from the mixture, and remained separated after solvent evaporation at room temperature (see Supplementary Fig. 2). Compounds with similar substituents but different numbers of hydroxyl groups were designed and compared as potential developers for the **WJRP**s, and their hydrochromic properties were investigated (Fig. 3a, right and see Supplementary Fig. 3a). The WJRPs based on the compounds with more adjacent hydroxyl groups were poorer to develop colour after introduction of water, allowing for lighter colour of WJRPs. This may be caused by the adjacent multi-hydroxyl groups in one molecule readily contributing to the intramolecular hydrogen bonding^32^, which weakens the interaction between the developers and ODB-2. *o*-Nitrophenol, as a developer, shows a poorer ability to develop colour for **WJRP** than *m*-nitrophenol and *p*-nitrophenol upon adding water, which also proves our speculation (see Supplementary Fig. 3b).

Based on the above experiments, phenol derivatives with one hydroxyl seem to be more suitable for developing colour of ODB-2 with the help of water. To investigate influence factors on developing colour of the binary system via water-promoted proton transfer between ODB-2 and developers, a series of phenol derivatives with various substituents has been explored on their competence as WJRPs’ developers. Firstly, effect of pK_a_, reflecting proton donating ability of developers^29,33^, on their water-developing colour intensities was investigated. After arranging them according to the colour intensity they developed (i.e., the reflectance value of WJRPs based on different developers at 586 nm after adding water) from weak to strong (see Supplementary Fig. 4), it was found that the reflectance was not fully in correlation with their pK_a_. Results indicate that other factors were also additionally affecting the ability of developers except for pK_a_. Considering ODB-2 is a lipophilic molecule with three planar substructure in its coloured state, both lipophilicity and planar structural affinity of the developer, which influences its intermolecular contact or distance with ODB-2, may also impact on their water-developing colour intensities. To verify this conjecture, a parameter of logP, referring to oil–water partition coefficient^34^, was introduced to evaluate the effect of lipophilicity and structural affinity of developers on developing colour for WJRP. Relationships between logP and abilities of four groups of phenol derivatives, divided with different structural backbones, to develop colour were investigated. As shown in Fig. 3b, in each group, hydrochromic colour contrast of WJRPs increased gradually with corresponding logP U.S. National Library of Medicine, https://chem.nlm.nih.gov/chemidplus/) of developers growing (see Supplementary Table 1), via increasing radius of substituted atoms (i.e., -F < -Br < -I) or extending lengths of substituted alkyl chains (i.e., from -CH_3_ to -C_4_H_9_). It indicates intermolecular contact or distance between developers and ODB-2 plays important roles in water-assisted colour intensities of their corresponding WJRPs via water-promoted proton transfer. This inference was proved further by the following studies. When intermolecular distance between ODB-2 and developers was shortened by raising concentration of both ODB-2 and B4H or increasing concentration of B4H alone in solution, the solution could turn to black without addition of water (see Supplementary Fig. 5). When intermolecular distance between ODB-2 and developers was increased via reducing concentration to a low level, the solution could not change colour even adding water (see Supplementary Fig. 6). Only when intermolecular distance between ODB-2 and developers or their concentration is proper, the original colourless solution containing ODB-2 and B4H would change its colour obviously with help of water (see Supplementary Fig. 7). To verify that intermolecular interaction between ODB-2 and developers made them get close (i.e., π–π stacking), naphthol which had a planar structure with fluorescence and might match well with upper planar structure of ODB-2, was used as a developer for WJRP. The fluorescence intensity of naphthol on the WJRP decreased drastically when introduction of ODB-2, and the reflectance of the WJRP was low to 20% indicating dark colour after addition of water (see Supplementary Fig. 8). This result suggests that various supramolecular interactions (i.e., hydrogen bonding, dipole–dipole interaction, π–π stacking, and Van der Waals forces, etc.) between developers and ODB-2, which are all deciding their distance for proton transfer, play major roles in developing colour intensity of the hydrochromic binary system.

PEG is crucial in the WJRP and plays multiple roles in the system. Firstly, as a passivation layer, PEG effectively block polyhydroxyl groups of the filter paper to keep ODB-2 in its colourless lactone form initially (see Supplementary Fig. 9). Secondly, PEG acts as a flexible-solid solvent and blocking agent for keeping dyes and developers in mono-dispersed-state and preventing proton transfer before introduction of water in the imaging layer. These functions were verified by the following facts. When loading the mixture of ODB-2 and B4H on glass with no polyhydroxyl groups, they exhibited colour in the absence of water. Once PEG was introduced into them, the mixture turned to colourless. After introduction of water, it turned to dark black immediately (Fig. 3c).

Water plays multi-funtions in this system. Firstly, it accelerates the proton transfer between developers and ODB-2 (Fig. 3d). Secondly, water’s dynamic network breaks the passivation effect of PEG on polyhydroxyl surroundings within filter paper, and facilitates the ring-opening of ODB-2 (Fig. 3e, blue line and see Supplementary Fig. 10). Thirdly, the dynamic and synergistic bound-water inside the system also helps to stabilize the coloured ring-open form of ODB-2. This conclusion was confirmed further by the observation that ODB-2 contained paper/solution turned to darker colour with water even without developers (Fig. 3e, red line and see Supplementary Fig. 11).

Taken together, the mechanism of the binary system based WJRP possessing hydrochromism was schematically summarized in Fig. 3f. In the absence of water, ODB-2 and developers got close enough due to their supramolecular interaction (blue dashed line in Fig. 3f), yet their proton transfer as well as stabilization effect of polyhydroxyls on open-form of ODB-2 was significantly inhibited by PEG to ensure the WJRP in colourless initially. After water was added, it broke through the initial blocking of PEG and linked up the hydroxyl groups of filter paper and the developers via its dynamic bound-water networks. Synergistic effect of polyhydroxyls and bound-water stabilized the zwitterion form of ODB-2 (dark black). When water evaporated, PEG recoveried to its position and provided interaction with developers and filter paper, respectively, rendering ODB-2 go back to its colourless lactone-form to realize colour-erasing.

### Water-jet printing on WJRP

**Fig. 4:**
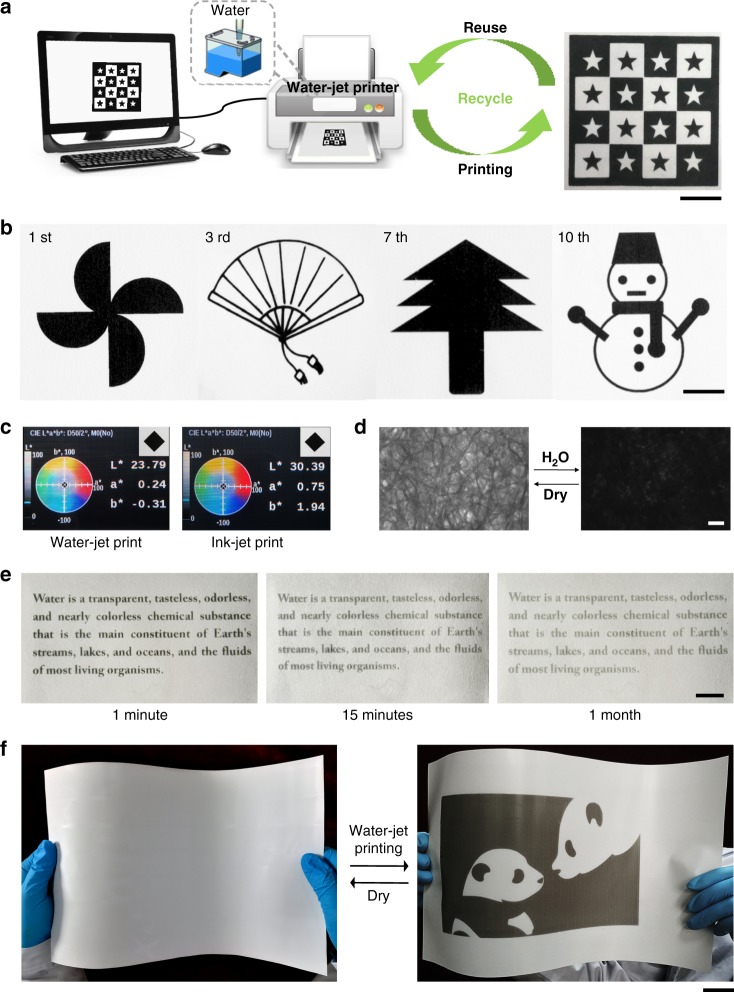
Repeatability, resolution, colour intensity and retaining time of water-jet prints. **a** Schematic illustrations of a printing-erasing cycle of WJRP printed with water-jet printer, scale bar = 1 cm. **b** Photographs of patterns printed on WJRP after 10 consecutive writing-erasing cycles (scale bar = 1 cm). **c** L*, a*, b* measured by spectrodensitometer and photographs of water-jet prints and ink-jet prints, respectively. **d** Microscopic images of WJRP before and after addition of water, scale bar = 200 µm. **e** Photographs of WJRP maintaining in ambient air after printing as time passed by, scale bar = 5 mm. **f** WJRP based on PET substrate before and after printed with water, scale bar = 2 cm

Application of the WJRP on the water-jet printing was explored next. To make the process of reversible printing more understandable, a schematic illustration was given in Fig. 4a. The WJRP is loaded to a water-jet printer tray and the designed image is input in a computer and printed by the water-jet printer, leading to appearance of colour on the paper. After completion of reading, the needless prints can be erased quickly by heating, and then the erased paper can be reused again by such reversible water-jet printing. To demonstrate the feasibility of the developed WJRP, various complicated patterns were printed on the rewritable paper. As shown in Fig. 4b, the water-jet prints have high contrast and excellent resolution. The third to the tenth rounds of prints all had good visibility and were comparable as the first round without contrast loss or any deficiency of details. We also printed the patterns with the same printer using traditional ink (hp704) to compare the print quality of WJRP with the commercial ink-jet printing. It is evident that the hue (a*, b*) and the colour intensity (L*) of the water-jet printed image on the binary system based WJRP is superior to the one obtained using conventional black ink (Fig. 4c and see Supplementary Fig. 12), which meets the standards of commercial products. The clear and uniform images on the WJRP after addition of water which are suitable for daily life is as a consequence of good dispersion of the molecules (i.e., B4H and ODB-2) on the paper (Fig. 4d).

Retaining time of water-jet prints is another important factor that limits the usage of WJRP. The current patterns printed by water can maintain about 15 min. To prolong the retaining time of the water-jet prints, polyvinyl alcohol (PVA) with spatial multi-hydroxyl was integrated into WJRP. As shown in Fig. 4e, the characters printed by water were still clearly readable after 7 days though the contrast decayed in certain range (see Supplementary Fig. 13). And WJRP with the remaining prints can be erased completely by wetting and heating at 80 °C simultaneously.

### WJRP using polyethylene terephthalate as substrate

To increase robustness, durability, and reversibility of WJRP, polyethylene terephthalate (PET) was selected to replace the filter paper as the substrate. Paper based on PET was prepared by a one-step simplified process, with coating the mixed solution of passivated agent and the binary system (see Supplementary Fig. 14a). The manufactured WJRP was colourless initially and the patterns on it appeared instantly after water-jet printing (Fig. 4f). Though the contrast of water-jet prints on PET is a little lower than that on filter paper, it is undoubtedly good enough for general reading and exhibits excellent reversibility (see Supplementary Fig. 14b–d). In addition, this WJRP can be generated easily via a roll to roll process with the PET substrate and to the size readily as we desired (see Supplementary Fig. 15), which offers great potential for converting the water-jet printing easily into realistic industrial production. According to a rough estimation, the cost per print of the WJRP with PET as the substrate would be approximately one-twentieth (based on consecutive 30 times reuse) of the normal ink-jet print (see Supplementary Table 2).

### WJRP with different colours

**Fig. 5:**
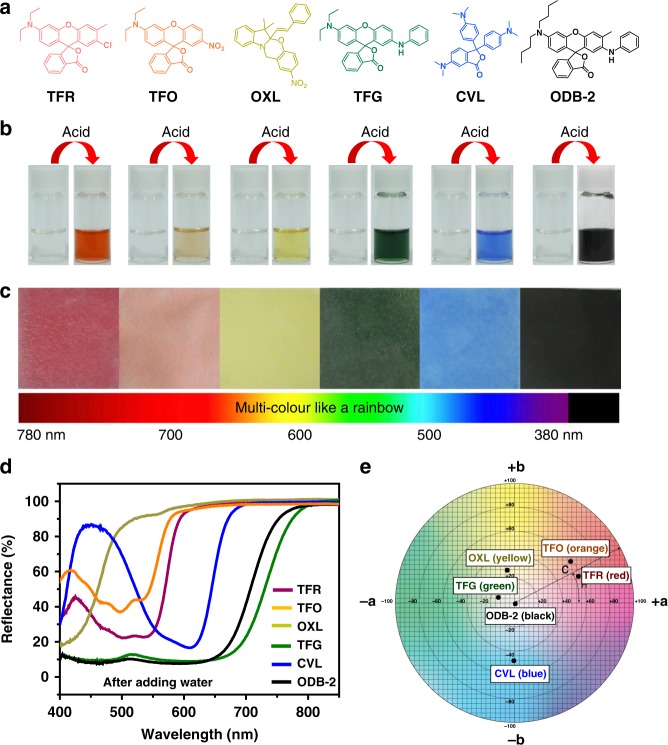
Other colours for WJRPs based on binary systems. **a** The structures of a series of acidochromic dyes. **b** Multicolours obtained by various acidochromic dyes in solution after treating with acid and **c** on solid state treated with B4H after addition of water and **d** corresponding UV-vis reflection spectra of the solid state. **e** Plots of corresponding positions of six kinds of WJRPs after adding water in the CIE 1931 colour space

In principle, this approach described herein should permit us to achieve various colour of WJRP by replacing ODB-2 with different acidochromic dyes. To explore the viability, three types of acidochromic leuco dyes were employed, that was fluorans (TFR, TFO, TFG) (the synthesis see Supplementary Methods), spirooxazines (OXL) and triarylmethanes (CVL) (Fig. 5a). They were colourless in acetonitrile (MeCN) and changed to red, orange, green, yellow, and blue, respectively after addition of acid (Fig. 5b). Nevertheless, these acidochromic dyes, which are similar to aforementioned ODB-2, all exibited their corresponding colours in a certain degree within the biomimetic binary systems based WJRPs after addition of water (Fig. 5c). These hydrochromic properties could not be attainable without the synergistic binary system on the paper (see Supplementary Fig. 16). It is worth noting that due to the similar structure of these dyes with ODB-2 (i.e., oleophilicity), B4H can also be proper developers for them all. High hydrochromic contrast ratio of over 50% of each WJRP was generated (Fig. 5d) and the colour of the WJRPs confirmed the possibilities of colours display in the whole visible spectrum. Moreover, the paper could convert to blank state when water evaporated, allowing the paper to be recycled (see Supplementary Fig. 17). To evaluate the quality of these coloured WJRPs, their L*, a*, b* value was presented in detailed in the supplementary information (see Supplementary Table 3). When plotting the positions of a portion of these colours in the CIE 1931 colour space^35^, the hue extent of these different WJRPs after addition of water on the CIE L*a*b* was relatively wide and scattered (Fig. 5e).

## Discussion

In summary, we broadened the scope of hydrochromic system from initial mono-dyes to a synergistic binary-system, and demonstrated an ingenious strategy toward developing environmental-friendly hydrochromic materials. The system, which supramolecularly bounded the acidochromic dyes with mild developers showed excellent hydrochromic performance under the long-anticipated bound-water manipulation with good sensitivity, high contrast and resolution, great reversibility and extended retention time (>1 month). In comparison to the known single-component hydrochromic materials, the binary system accomplished surmountable black WJRP and can easily expand to various hydrochromic colours within the entire visible range without complicated structural modification of known dyes. In addition, results indicated clearly that acidity/proton-donor-ability is not a critical factor to switch some acidochromic dyes, and mild acidic phenol group plays more roles to switch the pH-sensitive dyes and stabilize them with help of supramolecular interactions and adjacent bound-water. More importantly, such systems exhibited a unique property of biomimetic bound-water, which is ubiquitous in nature, that can surely enhance capability of mild proton donor (i.e. phenol) to induce change of molecular structure/colour via multi-molecules cooperative effect. Furthermore, our results reflect and support that tyrosine amino residue might play a critical role to switch various ion-channels of proteins and enzymatic bio-transformations with help of nearby bound-water. This work not only opens an avenue for development of various smart materials for practical applications, but also uncovers further, hopefully, the unique function of indistinct tyrosine residue of the enzymes/proteins in various biological transformations

## Methods

### Materials

3,5-Dimethylphenol (98%), 4-chloro-3-methylanisole (98%), 2-(4-diethylamino-2-hydroxybenzoyl) benzoic acid (98%) were purchased from Energy Chemical (Shanghai, China). Benzyl 4-hydroxybenzoate (98%) were purchased from TCI (Tokyo Chemical Industry) (Tokyo, Japan). Unless otherwise noted, all the other materials including developers were purchased from Aladdin Chemical (Shanghai, China), without further purification. Deionized water was purified by Milli-Q system. PEG 20000 (molecular weight: 17,000-22,000) was purchased from Guangfu Fine Chemical Research Institute (Tianjin, China). Cellulose filter paper (Whatman-Xinhua, grade 91, Hangzhou, China) is selected as the paper substrate. The origin of the graphics in Fig. 4 and Supplementary Fig. 14 were downloaded from https://image.baidu.com/ with permission of copyright holders.

### Measurements

Absorption spectra were measured using a Shimadzu UV-2550 PC double-beam spectrophotometer. Reflective UV–Vis spectroscopy of water-jet rewritable paper (WJRP) before and after addition of water and in situ kinetic measurement was tested via reflective mode of integrating sphere on Analitik Jena Specord®210 plus UV/VIS spectrophotometer, using BaSO_4_ as background, path length was 1 cm. The writing-erasing cycles experiment for WJR**P** was recorded by Maya 2000PRO fibre optical spectrometer with Ocean DH-2000-BAL UV-Vis-NIR light source. CIE L*, a*, b* was measured by X-rite spectrodensitometer. X-ray diffraction data was recorded on a Rigaku RAXIS-PRID diffractometer using the ω-scan mode with graphite monochromator Mo·Kα radiation (***λ*** = 0.71073 Å). ^1^H NMR (500 MHz) and ^13^C NMR (125 MHz) spectra were recorded on a Bruker AVANCE500 at room temperature. LC-HRMS analysis was performed on an Agilent 1290-micro TOF-Q II mass spectrometer.

### Preparation of WJRPs

The WJRP integrated with the binary system was prepared in a layer-by-layer manner. The filter paper substrate was coated with a layer of 10 wt% PEG aqueous solution and dried at 70 °C. Then DMF/H_2_O (10/1 by volume) solution of ODB-2 (0.02 M) and B4H (0.08 M) containing 1 wt% PEG is coated over the initial PEG layer. The methods used for the binary system of ODB-2 and B4H were used for the fabrication of WJRPs based on TFR and B4H, TFO and B4H, OXL and B4H, TFG and B4H as well as CVL and B4H with the same concentration.

### Preparation of WJRPs based on PET

The WJRP integrated with the binary system based on photographic paper was prepared by coating. The coating solution contains: PVP aqueous, surfactant 1292, surfactant FS-31, gelatin, the mixture of SiO_2_ and CaCO_3_ and the binary system of ODB-2 and B4H. Coat the paper with NO. 28 wire road and heat it in the oven at 40°C for white **WJRP**.

## Electronic supplementary material

Supplementary Information

## Data Availability

The data supporting the findings of this study are available from the corresponding authors on request. The source data underlying Fig. 3b, Supplementary Fig. 4 and Supplementary Table 1 is from the publically available source: U.S. National Library of Medicine. These data can be obtained free of charge via https://chem.nlm.nih.gov/chemidplus/chemidheavy.jsp.
