# Supplementary material for: Water assisted biomimetic synergistic process and its application in water-jet rewritable paper
Source: Nat Commun. 2018 Nov 16;9:4819. doi: 10.1038/s41467-018-07211-z (PMC6240070; doi:10.1038/s41467-018-07211-z)
Supplement: Supplementary file 1 — Supplementary Information [file 41467_2018_7211_MOESM1_ESM.pdf]

Supplementary Information

Water Assisted Biomimetic Synergistic Process and its Application in Water-Jet Rewritable Paper

Xi *et. al.*

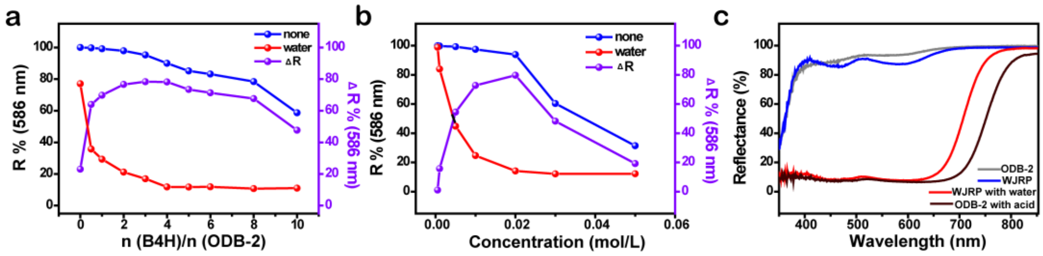

Supplementary Figure 1. Screen the optimal molar ratio of ODB-2 to benzyl 4-hydroxybenzoate (B4H) to achieve satisfactory black water-jet rewritable paper (WJRP) after addition of water. The different reflectance value of **WJRP**s at 586 nm integrated with (a) ODB-2 (0.02 M) with different molar ratio of B4H before and after addition of water. (b) ODB-2 and four equivalent of B4H in different concentration of ODB-2 (0.0005, 0.001, 0.005, 0.01, 0.02, 0.03, 0.05 M) before and after addition of water. (c) The UV-vis reflection spectra of **WJRP**s before and after addition of water and the same concentration of ODB-2 on the filter paper treated with PEG before and after addition of acid (1 M HCl).

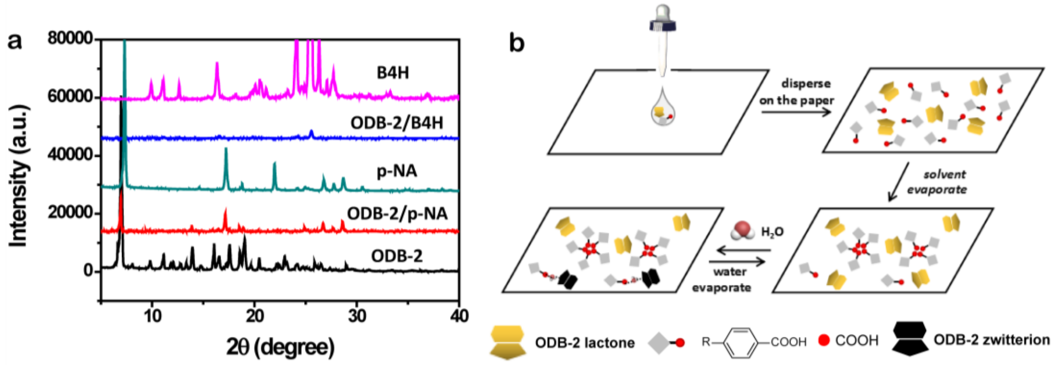

Supplementary Figure 2. Phase separation between ODB-2 and the carboxylic acid derivatives. (a) XRD patterns of crystalization of ODB-2, p-nitrobenzoic acid (p-NA), the mixture of ODB-2/p-NA (n/n 1/1), the mixture of ODB-2/B4H (n/n 1/1) and B4H. (b) Schematic representation of the carboxyl acids as developers for the colouration of **WJRP** before and after addition of water.

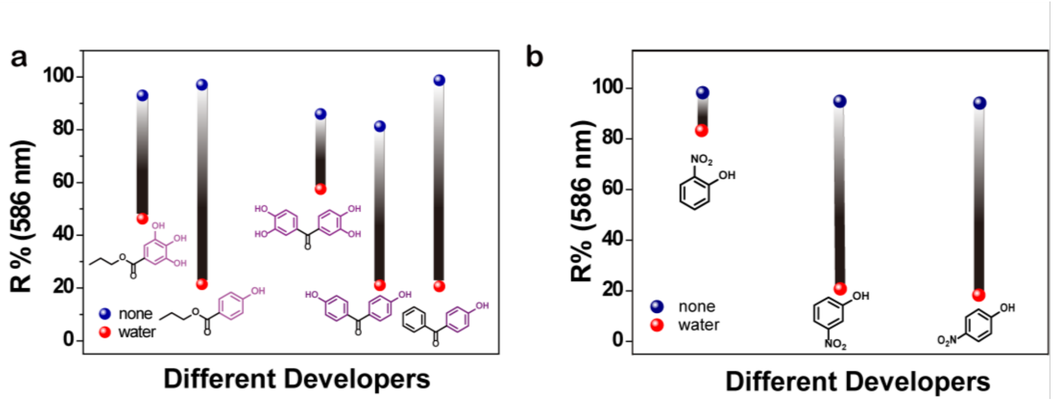

Supplementary Figure 3. Compounds with similar substituents but different numbers or positions of hydroxyl groups as developers. Variation of reflectance (R%) at 586 nm of the **WJRP**s integrated with ODB-2 and (a) different polyhydroxyl compounds (n/n 1/4) and (b) *o*-nitrophenol, *m*-nitrophenol and *p*-nitrophenol (n/n 1/4).

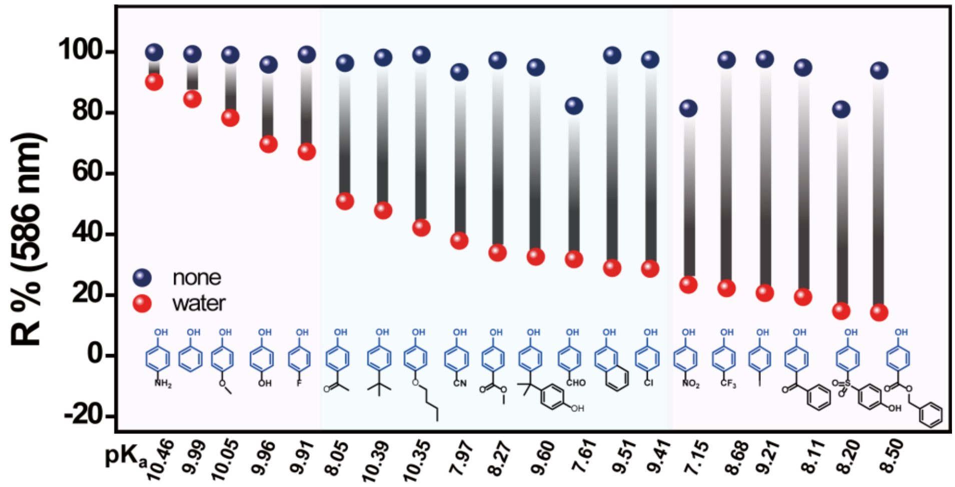

Supplementary Figure 4. Variation of reflectance (R%) at 586 nm of the WJRPs integrated with ODB-2 and a series of developers with different pKa (n/n 1/4).

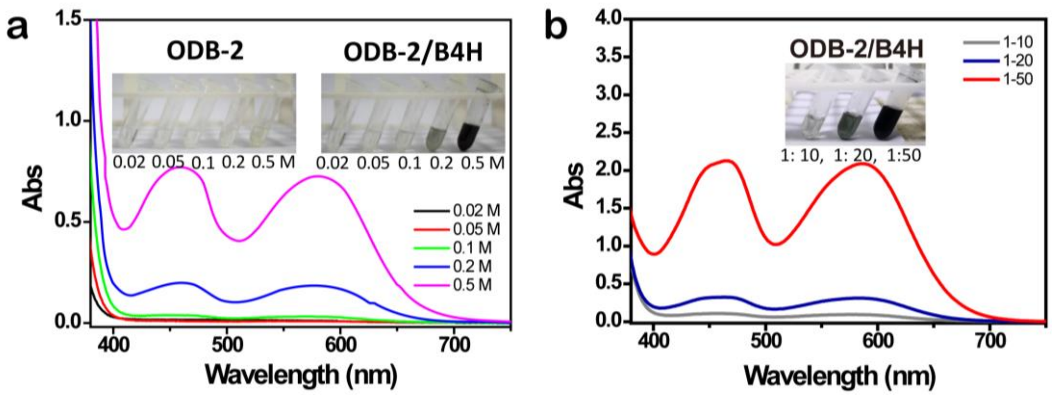

Supplementary Figure 5. Enhancing the interaction between ODB-2 and B4H by raising their concentration. (a) UV-vis absorption spectra and photographs (inset) of the solution of ODB-2 in DMF with different concentration before and after addition of four molar ratio amount of B4H, respectively. (b) Photographs and UV-vis spectra of the solution of ODB-2 (0.1 M) in DMF after addition of ten, twenty and fifty molar equivalent of B4H, respectively.

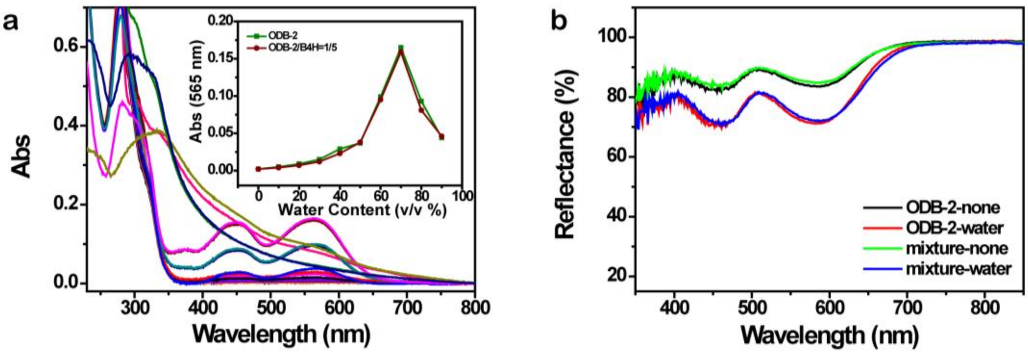

Supplementary Figure 6. Weakening the interaction between ODB-2 and B4H by decreasing their concentration. (a) UV-vis absorption spectra of ODB-2 and ODB-2/B4H (n/n 1/4) in variable mixtures of MeCN and water with increasing percentage of water by volume from 0 to 90% (2×10^{-5} M). Inset: Plots of variation of UV-vis absorption at 565 nm with increasing percentage of water by volume from 0 to 90%. (b) UV-vis reflection spectra of low concentration of ODB-2 (5×10^{-4} M) with and without four molar equivalent B4H on the filter paper before and after addition of water, respectively.

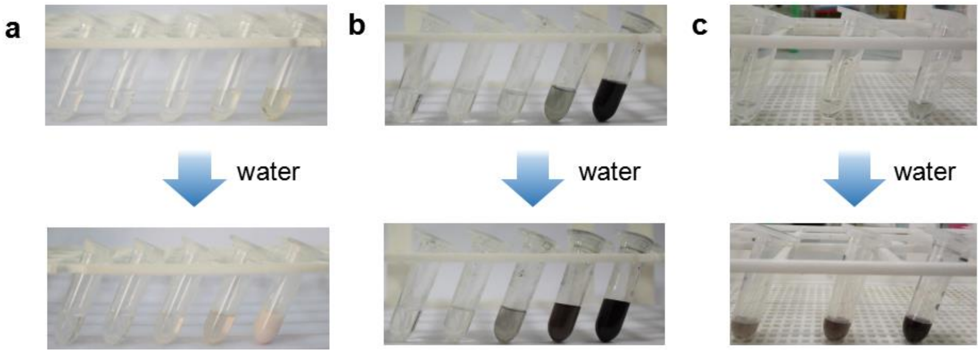

Supplementary Figure 7. ODB-2 and B4H in proper concentration change its colour with help of water. Photographs of (a) ODB-2 alone with different concentration (0.02, 0.05, 0.1, 0.2, 0.5 M) and (b) the binary system with different concentration of ODB-2 (0.02, 0.05, 0.1, 0.2, 0.5 M) and four molar equivalent B4H, respectively in DMF before and after addition of the same amount of water. (c) Photographs of ODB-2 (0.02 M) with different amounts of B4H (0.4 M, 1 M, 2M) in DMF before and after addition of the same amount water.

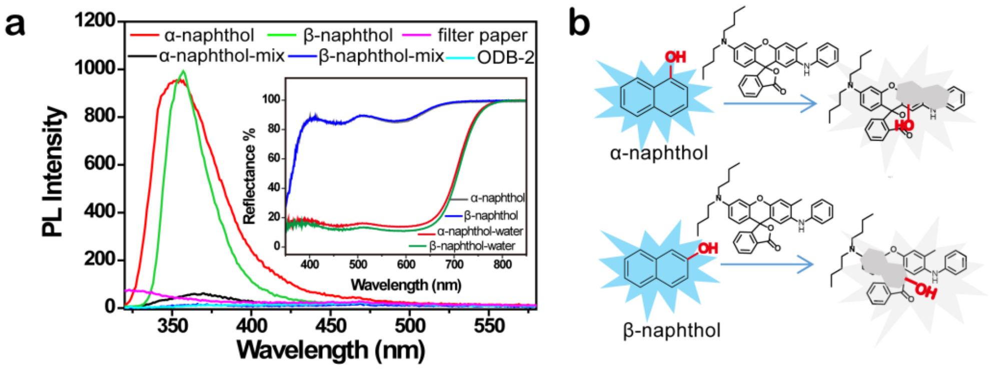

Supplementary Figure 8. Intermolecular interaction between ODB-2 and naphthol. (a) Fluorescent spectra of naphthol (0.08 M) alone, ODB-2 (0.02 M) alone and the mixture of ODB-2 (0.02 M) and naphthol (n/n 1/4) on the filter paper treated with PEG, respectively, excited by λ = 300 nm (slit: 3, 3). Inset: The UV-vis reflection spectra of **WJRP** based on the binary system of ODB-2 and naphthol before and after addition of water. (b) The proposed intermolecular interaction between ODB-2 and naphthol.

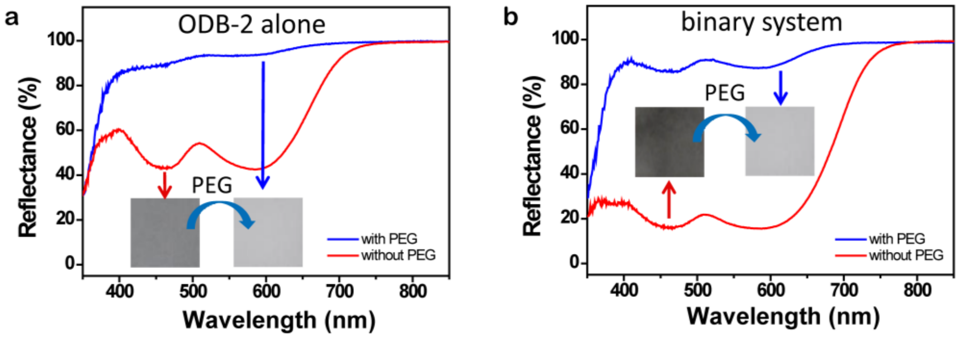

Supplementary Figure 9. Roles of polyethylene glycol (PEG) on WJRP. (a) UV-vis reflection spectra and photographs of ODB-2 (0.02 M) alone on the filter paper before and after introduction of PEG, respectively. (b) UV-vis reflection spectra and photographs of the mixture of ODB-2 (0.02 M) and B4H (n/n 1/4) before and after introduction of PEG, respectively.

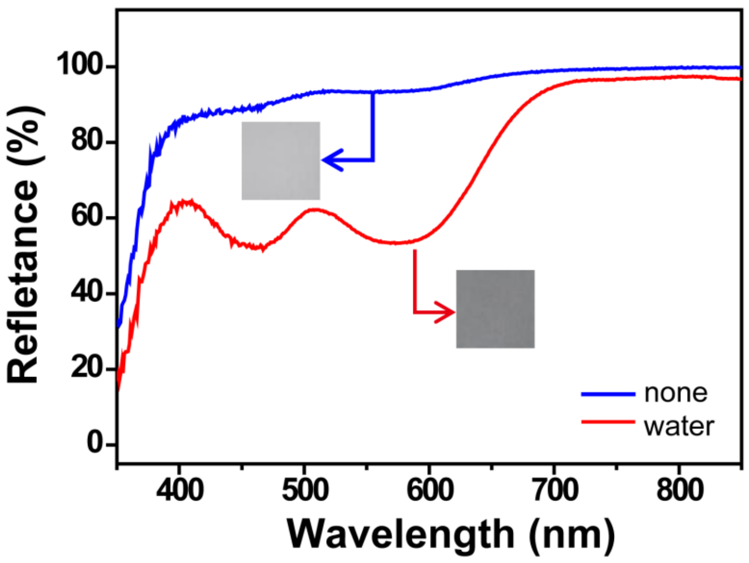

Supplementary Figure 10. UV-vis reflective spectra of ODB-2 on the filter paper treated with PEG before and after addition of water.

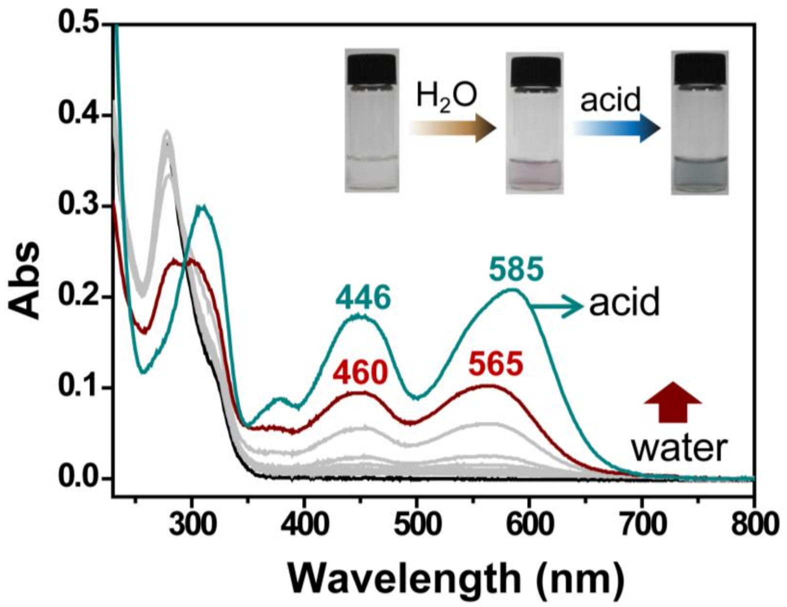

Supplementary Figure 11. Water-stimulated ODB-2 comparied with its halochromism. UV-vis absorption spectra of ODB-2 in variable mixtures of MeCN and water with increasing percentage of water by volume from 0 to 70% (1×10^{-5} M) and ODB-2 in MeCN/H_2O (v/v 3/7) with addition of CF_3COOH.

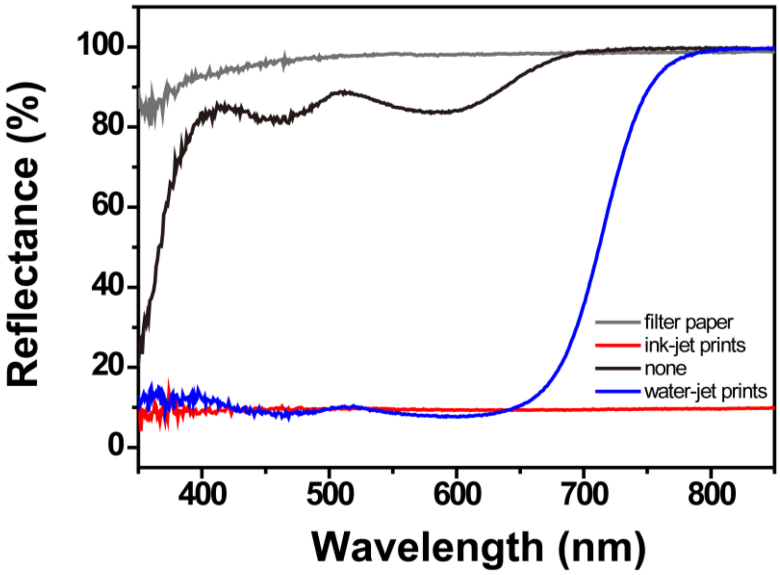

Supplementary Figure 12. UV-vis reflective spectra of water-jet prints on the binary system based WJRP compared with the conventional ink-jet prints on the filter paper.

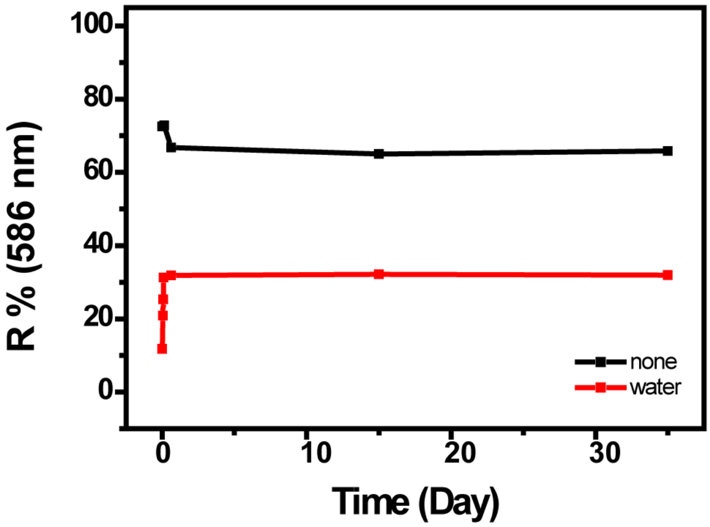

Supplementary Figure 13. Reflective spectra of retaining time of water-jet prints on WJRP. Time-dependent reflective visible spectra of the **WJRP** with PVA at 586 nm before (black dots) and after (red dots) addition of water.

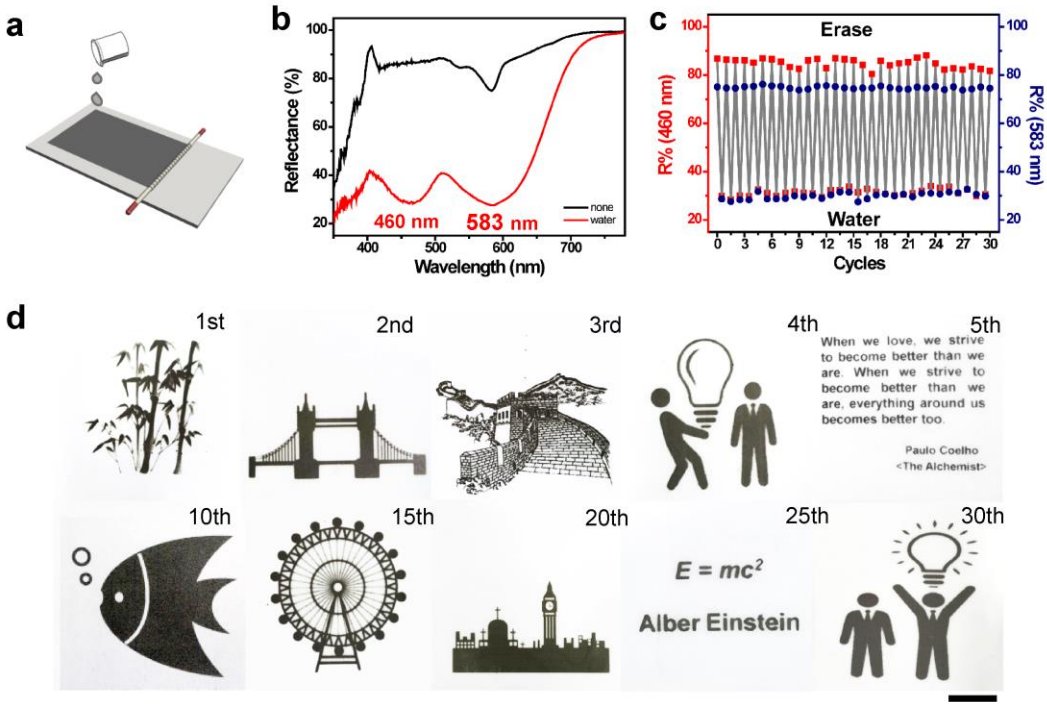

Supplementary Figure 14. UV-vis reflective spectra and repeatability of WJRP using polyethylene terephthalate (PET). (a) Schematic illustration of coating the **WJRP** based on PET. (b) The reflective UV-vis spectra of **WJRP** based on PET before and after addition of water. (c) Plots of the reflectivity at 460 nm (red dots) and 583 nm (blue dots), respectively versus the number of cycles as the **WJRP** using PET is cycled through water spraying (write) and water removal (erase). (d) Photographs of patterns and texts printed on **WJRP** using PET after 30 consecutive writing-erasing cycles (scale bar = 5 mm).

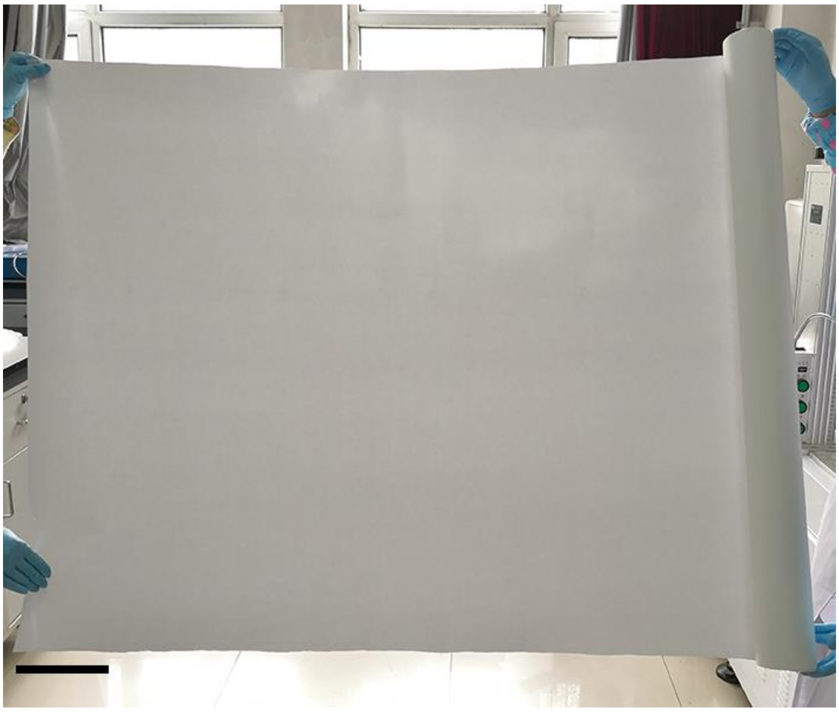

Supplementary Figure 15. The photograph of WJRP based on the substrate of PET prepared by a one-step method before addition of water. (scale bar = 10 cm)

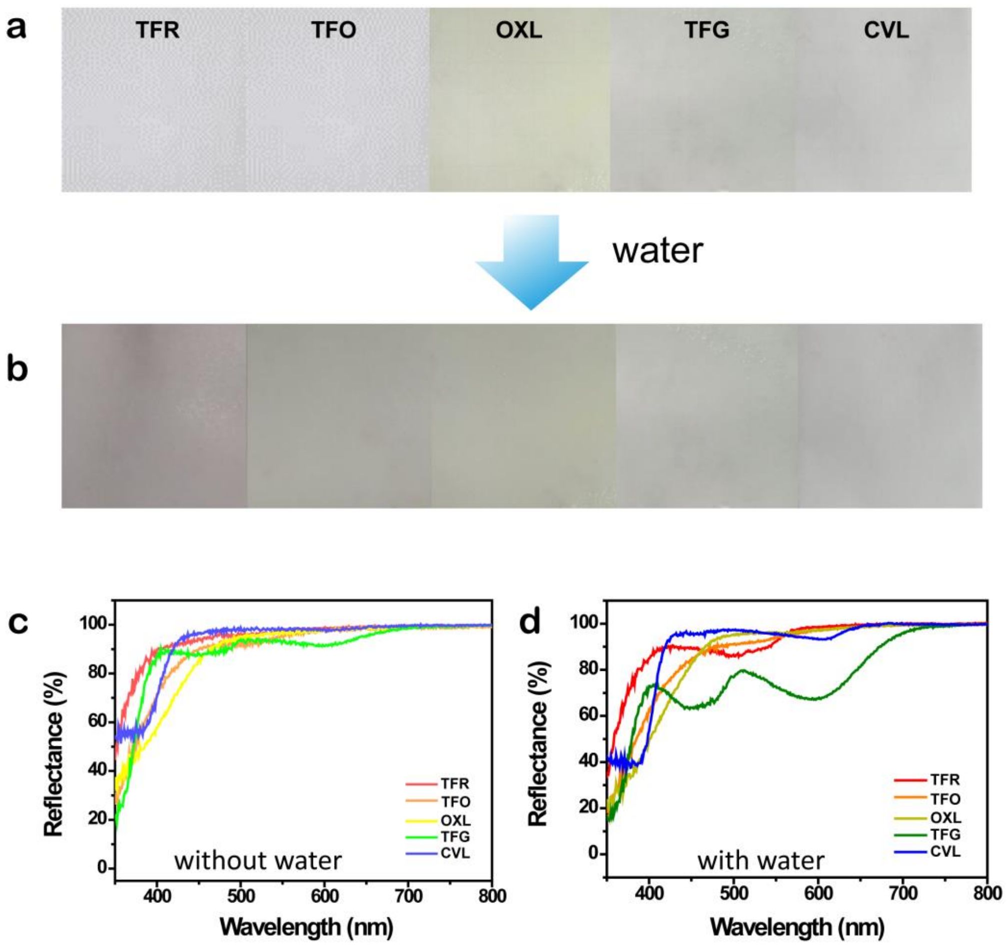

Supplementary Figure 16. WJRPs based on acidochromic dyes without developers. The photographs of a series of variable colour **WJRP**s based on acidochromic dyes without developers (a) before and (b) after addition of water. The UV-vis reflection spectra of **WJRP**s based on different acidochromic dyes alone (c) before and (d) after addition of water.

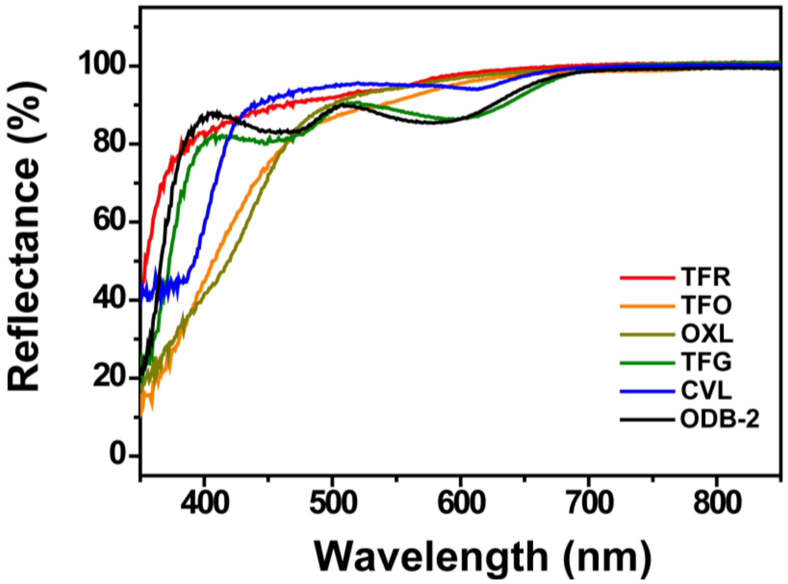

Supplementary Figure 17. The UV-vis reflection spectra of WJRPs based on different acidochromic dyes and B4H after addition of water and drying.

Supplementary Table 1. pK_a and LogP of developers with different substructure.[1]

R	F	Cl	I	OCH_3	OC_2H_5	OC_4H_9	$COCH_3$	COC_2H_5	COC_4H_9	$COOCH_3$	$COOC_2H_5$	$COOC_3H_8$	$COOC_4H_9$
pK_a	9.91	9.41	9.21	10.15	10.31	10.35	8.05	8.12	8.13	8.27	8.34	8.41	8.47
logP	1.77	2.39	2.91	1.58	1.81	2.90	1.35	2.03	2.93	1.96	2.47	3.04	3.57

Supplementary Table 2. The cost of water-jet printing and ink-jet printing.

	Water-jet printing			Ink-jet printing
Hydrochromic system	binary system		single molecule[2]	---
Substrate [a]	PET	filter paper	filter paper	printing paper
RMB ¥ [b]/sheet	0.2	0.1	0.1	0.04
ink	water	water	water	ink
RMB ¥/sheet	---	---	---	0.1[3]
Colouration components (Dosage/sheet)	ODB-2/B4H (0.006 g/0.014 g)	ODB-2/B4H (0.009 g/0.02 g)	hydrochromic fluoran dyes[2] (0.0002 g)	---
**Cost for colouration components RMB ¥/sheet**	0.0012+0.0029= **0.0041**	0.002+0.004 = **0.0060**	**0.00024**	---
Additives (Dosage/sheet)	PVP & Gelatin (0.056 & 0.073 g) DMF (0.4 mL)	PEG (0.05 g), PVA (0.2 g) DMF (4 mL)	PEG (0.3 g), PVA (0.2 g) EtOH (2 mL)	---
**Cost for additives RMB ¥/sheet**	0.00095+0.00058 +0.0028 = **0.0043**	0.0006+0.0016 +0.028 = **0.030**	0.0036+0.0016 +0.012 = **0.017**	---
Total cost for one paper (RMB ¥)	0.208	0.136	0.117	0.04
**Total printing cost for one print page.** Calculated based on reusing 30 times for PET sheet and 10 times for filter paper [c] **(RMB ¥)**	**0.0069**	**0.0136**	**0.0117**	**0.14** (printed on one side) **0.12** (printed on both sides)

Notes:

[a] The size of substrate is 21 × 29.7 cm^2 (A4).

[b] RMB ¥ is the abbreviation for Renminbi Yuan.

[c] The cost can be further cut down as the reuse increases.

Market bulk price for the materials:

ODB-2 and B4H in the hydrochromic binary system: 210 (RMB ¥) /1 kg;

Hydrochromic fluoran dyes in our previous work: 1200 (RMB ¥) /1 kg[2];

Polyethylene glycol (PEG, average Mn = 20000): 6 RMB ¥/500 g;

Polyvinyl alcohol (PVA): 4 RMB ¥/500 g;

Polyvinyl pyrrolidone (PVP): 8.5 RMB ¥/500 g;

Gelatin: 4 RMB ¥/500 g;

DMF: 3.5 RMB ¥/500 g;

EtOH: 3.2 RMB ¥/500 g.

Supplementary Table 3. L*, a*, b* values of WJRPs based on different acidochromic dyes before and after addition of water.

dyes	state	L*	a*	b*
TFR	none	90.38	0.97	1.75
	water	65.90	51.59	23.75
TFO	none	93.96	1.95	2.92
	water	67.28	44.13	34.19
OXL	none	94.33	-0.71	5.08
	water	89.6	-3.83	15.63
TFG	none	91.87	-1.37	3.82
	water	29.53	-8.74	3.44
CVL	none	93.19	-1.08	-0.05
	water	57.83	-0.68	-44.42
ODB-2	none	91.23	0.02	1.20
	water	23.79	0.24	-0.31

Supplementary Methods

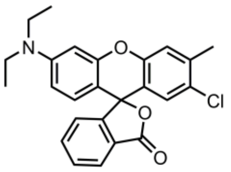

TFR was synthesized according to reported literature.[4] A mixture of 2-(4-diethylamino-2-hydroxybenzoyl) benzoic acid (0.63 g, 2 mmol) and 4-chloro-3-methylanisole (0.31 g, 2 mmol), H_2SO_4 (98%, 6 mL) was stired at room temperature for 30 min, then it was heated at 70°C for 7 h. The mixture was poured into ice water and filtered. The product was washed to neutral. The filter cake thus obtained was then refluxed with 20 % aq. sodium hydroxide (15 ml) and 20 ml toluene for 2 h. Extract the product from toluene. Then it was recrystallized from toluene to give light pink solid TFR (0.58 g, 70% yield). ^{1}H NMR (500 MHz, $CDCl_3$): δ (TMS, ppm): 8.03 (d, *J* = 7.5 Hz, 1H, Ar H), 7.67 (m, 1H, Ar H), 7.62 (m, 1H, Ar H), 7.18 (d, *J* = 7.5 Hz, 1H, Ar H), 7.14 (s, 1H, Ar H), 6.71 (s, 1H, Ar H), 6.57 (m, 1H, Ar H), 6.39 (m, 2H, Ar H), 3.36 (q, *J* = 7.0 Hz, 4H, CH_2), 2.38 (s, 3H, CH_3), 1.18 (t, *J* = 7.0 Hz, 6H, CH_3). LC-HRMS m/z: [*M* + H]$^+$ calcd for $C_{25}H_{22}ClNO_3$, 420.1288; found 420.1356.

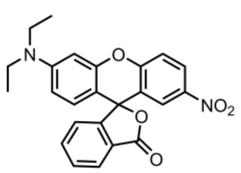

TFO was synthesized according to reported literature.[4] A mixture of 2-(4-diethylamino-2-hydroxybenzoyl) benzoic acid (0.31 g, 1 mmol) and *p*-nitrophenol (0.15 g, 1.1 mmol), H_2SO_4 (98%, 4 mL) were reacted at 80°C for 24 h. The mixture was poured into ice water and filtered. The product was washed to neutral. The filter cake thus obtained was then refluxed with 20 % aq. sodium hydroxide (10 ml) and 20 ml toluene for 2 h. Extract the product from toluene. The crude product was purified by column chromatography (CH_2Cl_2: MeOH = 40: 1, Rf = 0.4) to give yellow solid **TFO** (0.19 g, 45% yield). ^{1}H NMR (500 MHz, $CDCl_3$): δ (TMS, ppm): 8.26 (d, *J* = 9.0 Hz, 1H, Ar H), 8.08 (d, *J* = 9.0 Hz, 1H, Ar H), 7.7 (m, 3H, Ar H), 7.38 (d, *J* = 9.0 Hz, Ar H), 7.2 (d, *J* = 7.0 Hz, 1H, Ar H), 6.59 (d, *J* = 9.0 Hz, 1H, Ar H), 6.52 (s, 1H, Ar H), 6.43 (d, *J* = 8.0 Hz, 1H, Ar H), 3.38 (q, *J* = 7.0 Hz, 4H, CH_2), 1.19 (t, *J* = 7.0 Hz, 6H, CH_3). LC-HRMS m/z: [*M* + H]$^+$ calcd for $C_{24}H_{20}N_2O_5$ 417.1445, found 417.1441.

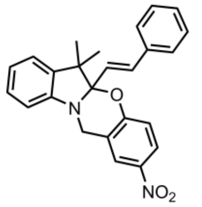

OXL was synthesized according to reported literature.[5] 1-(2-hydroxy-5-nitrobenzyl)-2,3,3-trimethylindoleninium chloride (0.51 mmol, 0.18 g) and benzaldehyde (0.5 mmol, 0.053 g) were refluxed in 10 ml ethanol solution for 3.5 h. Then the solvent was removed with rotary vacuum evaporator and treated with $NaHCO_3$ aqueous solution. Acetic ether was added to extract the product. Then the organic phase was separated and the solvent was distilled off under reduced pressure. The residue was recrystallized by acetic ether/hexane to get the product, yellow solid (0.13 g, 65%). ^{1}H NMR (300 MHz,

$CDCl_3$): δ (TMS, ppm): 8.08-7.79 (m, 2H, Ar H), 7.33 (m, 5H, Ar H), 7.10 (dd, *J* = 8.9, 6.6 Hz, 2H, Ar H), 6.89-6.84 (m, 2H, Ar H), 6.80 (d, *J* = 16.2 Hz, 1H, Ar H), 6.62 (d, *J* = 7.7, 1H, Ar H), 6.35 (d, *J* = 16.2 Hz, 1H, Ar H), 4.58 (s, 2H, CH), 1.53 (s, 3H, CH_3), 1.26 (s, 3H, CH_3). LC-HRMS m/z: $[M + H]^+$ calcd for $C_{25}H_{22}N_2O_3$ 399.1703, found 399.1700.

Supplementary References

[1] U.S. National Library of Medicine. [https://chem.nlm.nih.gov/chemidplus/chemidheavy.jsp].

[2] Xi, G.; Sheng, L.; Zhang, I.; Du, J.; Zhang, T.; Chen, Q.; Li, G.; Zhang, Y.; Song, Y.; Li, J.; Zhang, Y. M. & Zhang, S. X.-A. Endowing hydrochromism to fluorans via bioinspired alteration of molecular structures and microenvironments and expanding their potential for rewritable paper. *ACS Appl. Mater. Interfaces* **9**, 38032 (2017).

[3] Sheng, L.; Li, M.; Zhu, S.; Li, H.; Xi, G.; Li, Y.-G.; Wang, Y.; Li, Q.; Liang, S.; Zhong, K. & Zhang, S. X.-A. Hydrochromic molecular switches for water-jet rewritable paper. *Nat. Commun.* **5**, 3044 (2014).

[4] Meiqin, S. & Qiyu, T. Synthesis of fluoran dyes with improved properties. *Dyes Pigm.* **29**, 45 (1995).

[5] Deniz, E.; Tomasulo, M.; Sortino, S. & Raymo, F. M. Substituent effects on the photochromism of bichromophoric oxazines. *J. Phys. Chem. C* **113**, 8491 (2009).
